# TCS1, a Microtubule-Binding Protein, Interacts with KCBP/ZWICHEL to Regulate Trichome Cell Shape in *Arabidopsis thaliana*

**DOI:** 10.1371/journal.pgen.1006266

**Published:** 2016-10-21

**Authors:** Liangliang Chen, Yuancheng Peng, Juan Tian, Xiaohong Wang, Zhaosheng Kong, Tonglin Mao, Ming Yuan, Yunhai Li

**Affiliations:** 1 State Key Laboratory of Plant Cell and Chromosome Engineering, CAS Center for Excellence in Molecular Plant Sciences, Institute of Genetics and Developmental Biology, Chinese Academy of Sciences, China; 2 University of Chinese Academy of Sciences, China; 3 School of Life Science, Anhui Agricultural University, China; 4 State Key Laboratory of Plant Genomics, Institute of Microbiology, Chinese Academy of Sciences, China; 5 State Key Laboratory of Plant Physiology and Biochemistry, Department of Plant Sciences, College of Biological Sciences, China Agricultural University, China; University of Florida, UNITED STATES

## Abstract

How cell shape is controlled is a fundamental question in developmental biology, but the genetic and molecular mechanisms that determine cell shape are largely unknown. Arabidopsis trichomes have been used as a good model system to investigate cell shape at the single-cell level. Here we describe the *trichome cell shape 1* (*tcs1*) mutants with the reduced trichome branch number in Arabidopsis. *TCS1* encodes a coiled-coil domain-containing protein. Pharmacological analyses and observations of microtubule dynamics show that TCS1 influences the stability of microtubules. Biochemical analyses and live-cell imaging indicate that TCS1 binds to microtubules and promotes the assembly of microtubules. Further results reveal that TCS1 physically associates with KCBP/ZWICHEL, a microtubule motor involved in the regulation of trichome branch number. Genetic analyses indicate that *kcbp/zwi* is epistatic to *tcs1* with respect to trichome branch number. Thus, our findings define a novel genetic and molecular mechanism by which TCS1 interacts with KCBP to regulate trichome cell shape by influencing the stability of microtubules.

## Author Summary

The particular shape of plant cells is not only crucial for their biological functions but also affects the overall shape of organs. How cell shape is controlled is a fundamental question in developmental biology, and the study of plant cell shape regulation is an interesting part of plant biology. Arabidopsis trichomes have been used as a good model system to investigate cell shape at the single-cell level. In this study, we use Arabidopsis trichomes as a model to identify the *trichome cell shape 1* (*tcs1*) mutants with the reduced trichome branch number. *TCS1* encodes a microtubule binding protein, which is required for the stability of microtubules. We further find that TCS1 physically interacts with a microtubule motor involved in the regulation of trichome branch number. TCS1 acts genetically with this microtubule motor to control trichome branch number. Thus, our findings provide important insights into how the microtubule cytoskeleton determines cell shape.

## Introduction

The particular shape of plant cells not only relates to their functions but also influences the overall shape of organs. Arabidopsis trichomes are well established as a system for studying cell shape at the single-cell level [1–3]. Arabidopsis trichomes differentiate from single epidermal cells, which stop proliferating and begin endoreduplication cycle or endocycle. After three or four endoreduplication cycles, trichome cells have two successive branching events and morphological changes, and then form mature trichomes [1]. Trichomes on Arabidopsis leaves are regularly spaced and exhibit a distinctive shape with a stalk and three or four branches. The cytoskeletons appear to be important for establishing and maintaining the branching pattern of trichomes [4–6]. It is generally accepted that mutations in genes involved in the regulation of actin cytoskeleton often cause distorted trichomes, while the disruption of genes regulating the microtubule cytoskeleton usually influences the number of trichome branches [4,5,7–12]. However, the genetic and molecular mechanisms by which the cytoskeletons determine trichome cell shape remain largely unknown in plants.

In trichomes, microtubules, a major component of the plant cytoskeletons, not only regulate anisotropic cell expansion but also control cell branching. Several factors that regulate trichome branch number by influencing the microtubule cytoskeleton have been described in Arabidopsis. Arabidopsis TUBULIN FOLDING COFACTOR (TCF) C and TCFA have been suggested to be required for microtubule biogenesis, and their loss-of-function mutants show the reduced trichome branch number and shape as well as multiple growth defects [13,14], suggesting that the formation of new microtubules is likely to be important for the formation of new branches. KINESIN-13A has the microtubule-depolymerizing activity *in vitro* and *in vivo*, and *kinesin-13a* mutants produce trichomes with more branches [15]. Kinesin-like calmodulin-binding protein (KCBP/ZWICHEL) is involved in the regulation of microtubule stability and trichome morphogenesis in plants [4,16]. Trichomes on *kcbp*/*zwichel* (*zwi*) leaves have a short stalk and only one or two branches compared with wild-type trichomes with three or four branches [16]. KCBP-interacting Ca^2+^ binding protein (KIC) represses the activity of KCBP in response to Ca^2+^ and regulates trichome branching [17]. Plants overexpressing *KIC* produce trichomes with reduced branch number [17]. KCBP also physically interacts with ANGUSTIFOLIA (AN) in yeast cells, which is involved in the regulation of the microtubule cytoskeleton [18]. Trichomes on *an* leaves have one or two branches, indicating AN is required for normal trichome branching [18,19]. KCBP has been suggested to function with suppressors of *zwi* (SUZ) in a complex to control the number of trichome branches, but the *SUZ* genes remain to be cloned in Arabidopsis [20]. KCBP has also been recently reported to interact with both microtubules and F-actin to affect trichome branch initiation and elongation, respectively [21]. These studies imply that KCBP acts as an important node linking cytoskeletons with trichome cell shape.

To further understand the genetic and molecular mechanisms of cell shape control, we characterize *tcs1* mutants, which form trichomes with the reduced branch number. Mutations in *TCS1* influence the stability of microtubules. *TCS1* encodes a coiled-coil domain-containing protein, which binds to microtubules *in vitro* and *in vivo* and promotes the assembly of microtubules. Further results reveal that TCS1 interacts physically and genetically with KCBP/ZWI to control the number of trichome branches. Thus, our findings reveal a novel genetic and molecular mechanism of TCS1 and KCBP in trichome cell shape control.

## Results

### The *tcs1* mutants exhibit the reduced number of trichome branches

**Fig 1:**
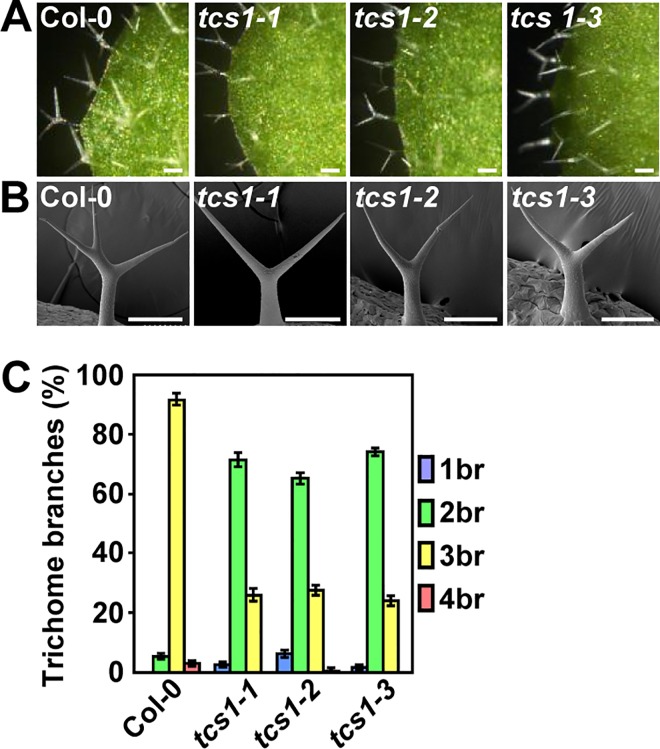
*tcs1* mutants show the reduced number of trichome branches. (A) Light microscope images of Col-0, *tcs1-1*, *tcs1-2* and *tcs1-3* trichomes. Bars = 100 μm. (B) Scanning electron microscope images of Col-0, *tcs1-1*, *tcs1-2* and *tcs1-3* trichomes. Bars = 100 μm. (C) Trichome branch (br) distribution of Col-0, *tcs1-1*, *tcs1-2* and *tcs1-3* first pair of leaves at 15 days after germination (DAG). Values are given as mean ± SE.

We isolated the *trichome cell shape 1* (*tcs1*) mutants in a screen of publicly available T-DNA mutant collections of *Arabidopsis thaliana*. The *tcs1-1*, *tcs1-2* and *tcs1-3* trichomes had the reduced branch number compared with wild-type trichomes (Fig 1). By contrast, the *tcs1* mutants did not show any obvious defects in plant growth. Progeny of crosses of the three lines indicated that they are allelic. We further measured the number of trichome branches using the first pair of leaves. In wild-type leaves, trichomes normally had two branching points with three branches (92%), although trichomes with four branches were occasionally found (Fig 1C). By contrast, about 70% and 25% of trichomes on *tcs1* leaves had two and three branches, respectively (Fig 1C). The tips of *tcs1* trichome branches were sharp, as those observed in wild-type trichome branches (Fig 1B). Thus, these results show that *TCS1* influences the number of trichome branches in Arabidopsis.

### Trichomes of *tcs1* are hypersensitive to the microtubule-disrupting drug oryzalin and the microtubule-stabilizing drug paclitaxel

**Fig 2:**
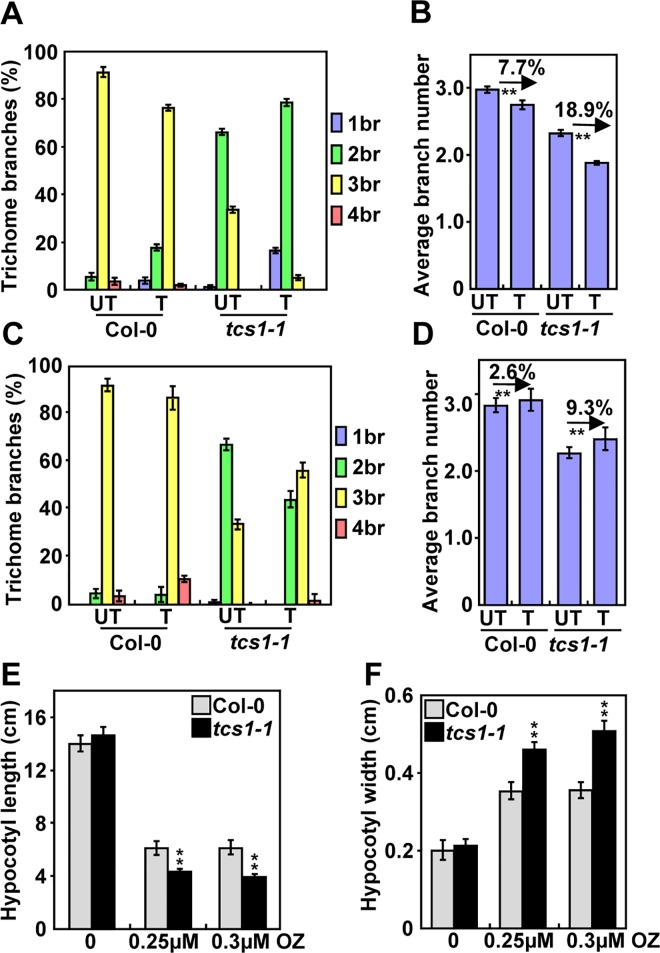
Trichomes and hypocotyls of *tcs1-1* are hypersensitive to the microtubule-disrupting drug oryzalin and the microtubule-stabilizing drug paclitaxel. (A) Trichome branch distribution of Col-0 and *tcs1-1* treated with (T) or without (UT) 20 μM oryzalin for 2 hours. The branch number of Col-0 and *tcs1-1* trichomes was examined after a 10-day recovery on ½ MS medium. (B) The average number of Col-0 and *tcs1-1* trichome branches treated with (T) or without (UT) 20 μM oryzalin for 2 hours. The branch number of Col-0 and *tcs1-1* trichomes was examined after a 10-day recovery on ½ MS medium. (C) Trichome branch distribution of Col-0 and *tcs1-1* treated with (T) or without (UT) 20 μM paclitaxel for 2 hours. The branch number of Col-0 and *tcs1-1* trichomes was examined after a 10-day recovery on ½ MS medium. (D) The average number of Col-0 and *tcs1-1* trichome branches treated with (T) or without (UT) 20 μM paclitaxel for 2 hours. The branch number of Col-0 and *tcs1-1* trichomes was examined after a 10-day recovery on ½ MS medium. (E) The average hypocotyl length of Col-0 and *tcs1-1* seedlings grown in ½ MS containing 0 μM, 0.25 μM and 0.3 μM oryzalin (OZ) for 15 days in dark. (F) The average hypocotyl width of Col-0 and *tcs1-1* seedlings grown in ½ MS containing 0 μM, 0.25 μM and 0.3 μM oryzalin (OZ) for 15 days in dark. Values (A-F) are given as mean ± SE. **P<0.01 compared with the wild type (Student’s *t* test).

In Arabidopsis, the reduced branch number of trichomes is often correlated with a decrease in the level of endoreduplication or the destabilization of microtubules [18,22]. We firstly investigated whether *TCS1* could affect endoreduplication in trichome cells. As the nuclear size is often associated with the ploidy level, we measured the nuclear size of Col-0 and *tcs1-1* trichomes. The average nuclear size of *tcs1-1* trichomes was similar to that of wild-type trichomes (S1A and S1B Fig). The ploidy levels in *tcs1-1* leaves were comparable with those in wild-type leaves (S1 Fig). These results suggest that *TCS1* may not regulate endoreduplication. We then asked whether *TCS1* could influence the microtubule cytoskeleton. The microtubule-disrupting drug oryzalin has been shown to destabilize microtubules, leading to a decrease in the number of trichome branches in Arabidopsis [23]. We therefore treated 4-day-old seedlings of Col-0 and *tcs1-1* with 20 μM oryzalin for 2 hours. After a 10-day recovery on ½ MS medium, we examined the branch number of Col-0 and *tcs1-1* trichomes. As shown in Fig 2A and 2B, the oryzalin treatment caused a 7.7% decrease in the average number of Col-0 trichome branches, while the oryzalin treatment resulted in an 18.9% reduction in the average number of *tcs1-1* trichome branches. The microtubule-stabilizing drug paclitaxel (taxol) has been reported to stabilize microtubules [23]. We asked whether taxol could rescue the trichome branch phenotype of *tcs1*. Four-day-old seedlings of Col-0 and *tcs1-1* were treated with 20 μM taxol for 2 hours. After a 10-day recovery on ½ MS medium, we examined the branch number of Col-0 and *tcs1-1* trichomes. In our growth condition, the taxol treatment caused a 2.6% increase in the average number of Col-0 trichome branches, while the taxol treatment resulted in a 9.3% increase in the average number of *tcs1-1* trichome branches (Fig 2C and 2D), suggesting that taxol partially rescues the phenotype of *tcs1-1* trichome branches.

As the microtubule is crucial for hypocotyl elongation [24], we asked whether TCS1 affects hypocotyl growth. As shown in Fig 2E and 2F, the average length and width of dark-grown *tcs1-1* hypocotyls was comparable with that of dark-grown Col-0 hypocotyls. We then treated dark-grown Col-0 and *tcs1-1* seedlings with oryzalin and measured their hypocotyl length and width. After oryzalin treatment, hypocotyls of *tcs1-1* were significantly shorter and wider than those of the wild type (Fig 2E and 2F). Epidermal cells in *tcs1-1* hypocotyls were short and wide in comparison with those in wild-type hypocotyls (S2 Fig). These results show that hypocotyls of *tcs1-1* are hypersensitive to oryzalin treatment than wild-type hypocotyls.

### Disruption of *TCS1* influences the stability of microtubules

**Fig 3:**
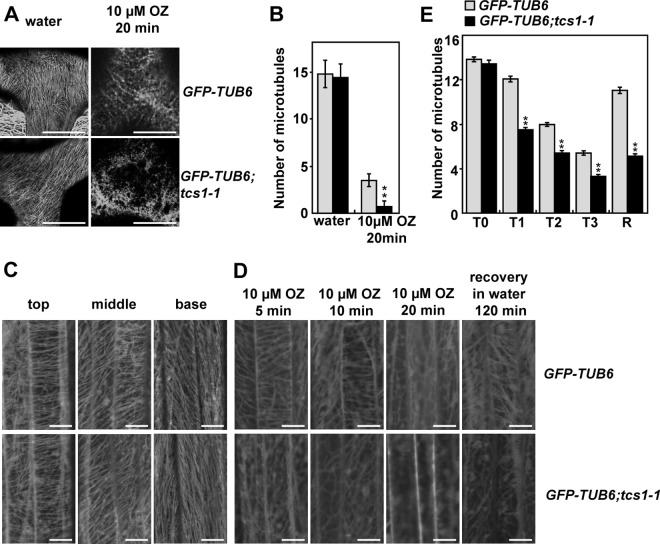
Disruption of *TCS1* influences the stability of microtubules in trichomes. (A) Cortical microtubules in trichome cells of *GFP-TUB6* and *GFP-TUB6;tcs1-1* treated with water or 10 μM oryzalin (OZ) for 20 minutes. Bars = 20 μm. (B) The microtubule density at the branch junction of *GFP-TUB6* and *GFP-TUB6;tcs1-1* trichome cells treated with water or 10 μM oryzalin for 20 minutes. ImageJ software was employed to quantify the numbers of cortical microtubules. A line of fixed length (10 μm) perpendicular to the orientation of the most cortical microtubules at the trichome branch junction was drawn, and the number of cortical microtubules across the line was counted. At least 10 cells from each treatment were used, and four lines of fixed length were drawn for each cell. Values are given as mean ± SE. **P<0.01 compared with the wild type after oryzalin treatment (Student’s *t* test). (C) Cortical microtubules in epidermal cells of *GFP-TUB6* and *GFP-TUB6;tcs1-1* etiolated hypocotyls. *GFP-TUB6* and *GFP-TUB6;tcs1-1* seedlings were grown in dark for 72 hours. Epidermal cells in the top, middle and basal regions of *GFP-TUB6* and *GFP-TUB6;tcs1-1* hypocotyls were observed by confocal microscopy. Bars = 10 μm. (D) Cortical microtubules in epidermal cells in the middle region of *GFP-TUB6* and *GFP-TUB6;tcs1-1* etiolated hypocotyls treated with 10 μM oryzalin (OZ) for 5, 10 and 20 minutes (min). Oryzalin was then washed off, and cortical microtubules were imaged after 2 hours. Bars = 10 μm. (E) Quantification of cortical microtubules in hypocotyl epidermal cells of *GFP-TUB6* and *GFP-TUB6;tcs1-1* seedlings treated with 10 μM oryzalin for 0 min (T0), 5 min (T1), 10 min (T2) and 20 min (T3), respectively (n > 30 cells for each sample). The R represents that oryzalin was then washed off for 2 hours. The y axis represents the number of cortical microtubules across a fixed line (20 μm) vertical to the orientation of most cortical microtubules in the cell. Values are given as mean ± SE. **P<0.01 compared with *GFP-TUB6* (Student’s *t* test).

As *tcs1* trichomes had the reduced branch number and were hypersensitive to oryzalin and taxol, we asked whether *TCS1* affects the stability of microtubules in trichome cells. We therefore crossed *GFP-TUB6* transgenic plants with the *tcs1-1* mutant and generated *GFP-TUB6;tcs1-1* plants. Cortical microtubule arrays in *tcs1-1* trichome cells were similar to those in wild-type trichome cells (Fig 3A). We then applied the microtubule-disrupting drug oryzalin to trichome cells of *GFP-TUB6* and *GFP-TUB6;tcs1-1* leaves. As shown in Fig 3A and 3B, cortical microtubule arrays disappeared faster in *tcs1-1* trichome cells than those in wild-type trichome cells. We counted the number of cortical microtubules in the trichome branch junction. Microtubules were similar in density before oryzalin treatment. However, more cortical microtubules were disrupted in *tcs1-1* than in the wild type after drug treatment. These results indicate that *TCS1* influences the stability of microtubules in trichomes. Similarly, we observed that microtubule arrays disappeared relatively faster in epidermal cells of *tcs1-1* cotyledons than those in epidermal cells of wild-type cotyledons after oryzalin treatment (S3 Fig).

As *tcs1* hypocotyls were hypersensitive to the microtubule-disrupting drug oryzalin, we investigated whether *TCS1* is required for the stability of microtubules in hypocotyl cells. Cortical microtubule arrays in epidermal cells of *GFP-TUB6*;*tcs1* hypocotyls were comparable with those of *GFP-TUB6* hypocotyls (Fig 3C and 3E). We then applied the microtubule-disrupting drug oryzalin to epidermal cells of etiolated *GFP-TUB6* and *GFP-TUB6;tcs1-1* hypocotyls. Cortical microtubule arrays disappeared relatively faster in epidermal cells of *tcs1-1* hypocotyls than those in epidermal cells of wild-type hypocotyls (Fig 3D and 3E). When oryzalin was washed off after the treatment, the recovery of cortical microtubules in epidermal cells of *tcs1-1* hypocotyls was slower than that in epidermal cells of wild-type hypocotyls (Fig 3D and 3E). Taken together, these results indicate that *TCS1* influences the stability of microtubules.

### *TCS1* encodes a coiled-coil domain-containing protein

**Fig 4:**
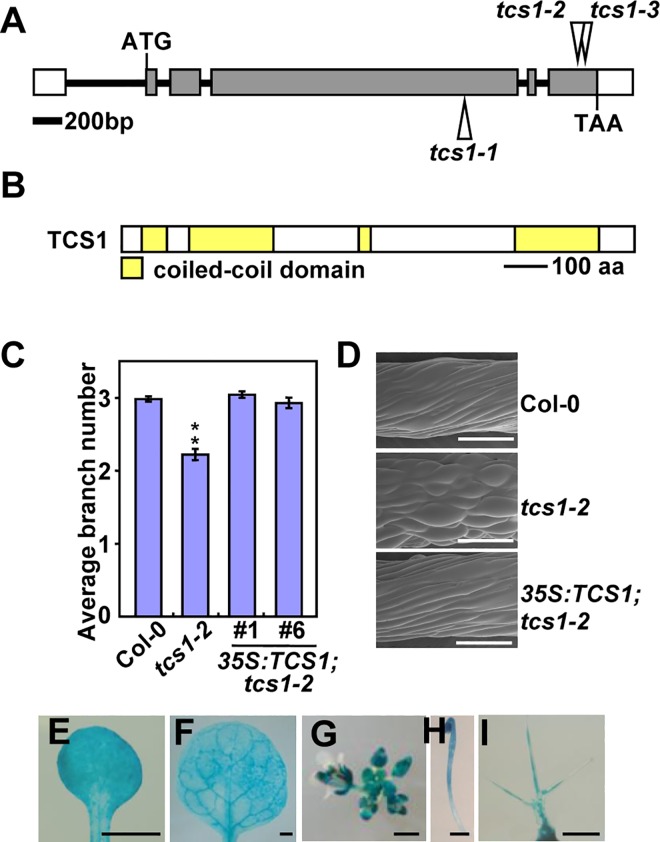
Identification of the *TCS1* gene. (A) The *TCS1* gene structure. The start codon (ATG) and the stop codon (TAA) are indicated. Closed boxes indicate the coding sequences, open boxes show the 5' and 3' untranslated regions, and the line between boxes indicates the intron. The T-DNA insertion sites (*tcs1-1*, *tcs1-2* and *tcs1-3*) in *TCS1* are shown. (B) The predicted TCS1 protein contains four coiled-coil domains. aa, amino acids. (C) The average number of trichome branches in Col-0, *tcs1-2*, *35S*:*TCS1;tcs1-2#1* and *35S*:*TCS1*;*tcs1-2#6* leaves. Values are given as mean ± SE. **P<0.01 compared with Col-0 (Student’s *t* test). (D) Scanning electron microscope images of epidermal cells in Col-0, *tcs1-2* and *35S*:*TCS1;tcs1-2* hypocotyls grown in ½ MS containing 0.3 μM oryzalin for 15 days in dark. Bars = 200 μm. (E-I) *TCS1* expression activity was monitored by *pTCS1*:*GUS* transgene expression. Histochemical analysis of GUS activity in a cotyledon (E), a leaf (F), an inflorescence (G), a 4-day-old dark-grown seedling (H) and a trichome (I). Bars = 1 mm in (E-H) and 0.1 mm in (I).

The *tcs1-1*, *tcs1-2* and *tcs1-3* mutants were identified from the T-DNA insertions in the fourth exon and the sixth exon of the gene *At1g19835*, respectively (Fig 4A). T-DNA insertions were confirmed using T-DNA specific and flanking primers (S4A–S4C Fig). We further investigated the expression level of *At1g19385* in *tcs1-1*, *tcs1-2* and *tcs1-3* mutant seedlings. As shown in S4D Fig, the full length transcript of *At1g19835* was not detected in *tcs1* mutants, suggesting that *tcs1* mutants are loss-of-function alleles. A plasmid containing wild-type *At1g19835* cDNA driven by a 35S promoter was introduced into the *tcs1-2* mutant. Transgenic plant exhibited complementation of *tcs1-2* phenotypes (Fig 4C and 4D). In addition, transformation of *tcs1-1* with TCS1-GFP fusion protein under the control of the *TCS1* promoter (*pTCS1*:*TCS1-GFP*) restored a wild-type phenotype (S4E Fig). Therefore, these results indicate that *At1g19835* is the *TCS1* gene.

*TCS1* encodes a 982-amino-acid protein that contains four coiled-coil domains, which belongs to a family of long coiled-coil protein that consists of 7 members in Arabidopsis [25] (Fig 4B; S5 Fig). Although the family members have been named as filament-like plant proteins (AtFPP), their biochemical and biological functions are totally unknown in Arabidopsis [25]. By performing a BLAST search in the databases, we identified TCS1 homologs in *Brassica rapa*, *Gossypium raimondii*, *Sorghum bicolor*, *Zea mays*, and *Oryza sativa*, but we did not find convincing homologs from animals and yeasts (S5 Fig), suggesting that TCS1 and its homologs might have evolved to control cell morphogenesis in plants.

To determine the expression pattern of *TCS1*, RNA from roots, flowers, seedlings and leaves were investigated by RT-PCR analysis. *TCS1* mRNA was detected in all plant organs tested (S6 Fig). Tissue-specific expression pattern of *TCS1* was examined using histochemical assay of GUS activity of transgenic plants containing the *TCS1* promoter:*GUS* fusion (*pTCS1*:*GUS*). GUS activity was detected in cotyledons, leaves, inflorescences and developing etiolated hypocotyls (Fig 4E–4H). GUS activity was also observed in trichomes (Fig 4I), consistent with the role of *TCS1* in trichome morphogenesis.

### TCS1 binds to microtubules and promotes microtubule assembly

**Fig 5:**
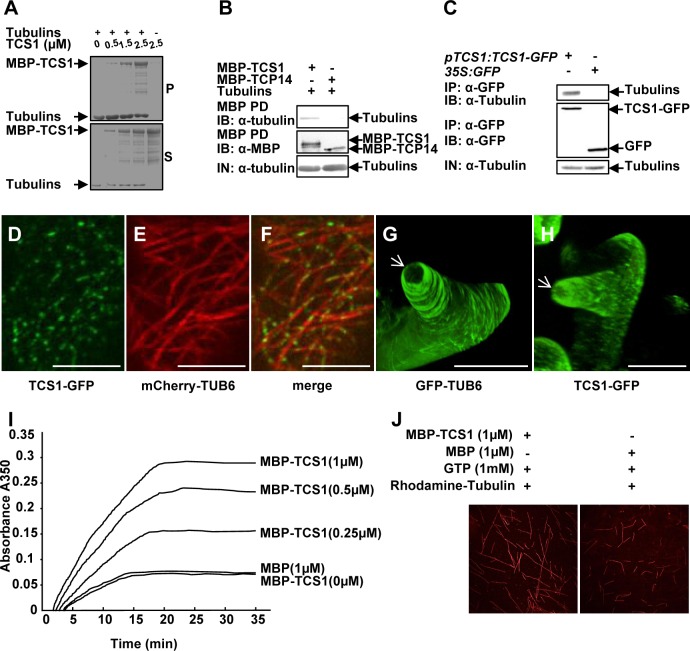
TCS1 binds to microtubules and promotes microtubule assembly. (A) MBP-TCS1 fusion protein was cosedimented with paclitaxel-stabilized microtubules prepolymerized from 8 μM tubulins. After high-speed centrifugation, the amount of MBP-TCS1 in pellets (P) increased when higher concentrations of MBP-TCS1 proteins were added. MBP-TCS1 mostly appeared in the supernatant (S) in the absence of tubulins. (B) TCS1 directly interacts with tubulins *in vitro*. Tubulins were pulled down (PD) by MBP-TCS1 immobilized on amylose resin and analyzed by immunoblotting (IB) using an anti-tubulin antibody. MBP-TCP14 was used as a negative control.(C) TCS1 interacts with tubulins in Arabidopsis. Total proteins from *pTCS1*:*TCS1-GFP* and *35S*:*GFP* transgenic plants were immunoprecipitated (IP) with GFP-Trap-A, and the immunoblot (IB) was detected with anti-GFP and anti-tubulin antibodies, respectively. Tubulins were detected in the immunoprecipitated GFP-TCS1 complex but not in the control (GFP). (D-F) TCS1 colocalizes with cortical microtubules (MTs) in Arabidopsis epidermal pavement cells. TCS1-GFP (D), mCherry-TUB6 (E) and merged (F) images are shown. TCS1-GFP localizes along cortical microtubules (mCherry-TUB6) in a punctate pattern. Scale bars = 5 μm. (G and H) GFP fluorescence of GFP-TUB6 (G) and TCS1-GFP (H) in elongating trichome branches. The white arrows indicate the three dimension (3-D) reconstruction of the microtubule-depleted zone at the extreme apex of elongating trichome branches. Bars = 20 μm.(I) MBP-TCS1 promotes the assembly of microtubules. The time course of tubulin polymerization from 20 μM tubulins in the presence of different concentrations of MBP-TCS1 was detected turbidimetrically by absorbance at 350 nm. 1μM MBP was used as a negative control. (J) Confocal microscopy analysis showed that TCS1 promotes tubulin assembly *in vitro*. Rhodamine labeled tubulins (20 μM) were incubated with 1 μM MBP-TCS1 and 1 μM MBP at 37°C for 30 min, respectively. Microtubules were visualized under confocal microscopy.

As *tcs1* affects the stability of microtubules, we asked whether TCS1 could directly bind to the microtubules. A cosedimentation assay was used to analyze the binding of TCS1 to taxol-stabilized microtubules. TCS1 was expressed as a maltose binding protein (MBP) fusion protein (MBP-TCS1) in *E*.*coli*. As shown in Fig 5A and S7A Fig, MBP-TCS1 was cosedimented with the microtubules. The binding of TCS1 to microtubules was saturated at a stoichiometry of about 0.38 M MBP-TCS1 per mole of tubulin dimers (S7C Fig). The binding of the positive control (AUGMIN subunit 8, AUG8) to microtubules was saturated at a stoichiometry of about 0.22 M His-AUG8 per mole of tubulin dimers in our experimental conditions (S7B and S7C Fig) [26]. We then asked whether TCS1 could directly interact with tubulins. To test this, we conducted pull-down experiments. As shown in Fig 5B, MBP-TCS1 bound to tubulins, while the negative control (MBP-TCP14) did not interact with tubulins. Thus, these results indicate that TCS1 physically interacts with tubulins *in vitro*.

We further performed co-immunoprecipitation analyses to detect the interaction of TCS1 with tubulins in Arabidopsis. Total proteins from *pTCS1*:*TCS1-GFP* or *35S*:*GFP* plants were isolated and incubated with GFP-Trap-A agarose beads to immunoprecipitate TCS1-GFP and GFP. The anti-GFP and anti-tubulin antibodies were used to examine immunoprecipitated proteins, respectively. As shown in Fig 5C, tubulins were found in the immunoprecipitated TCS1-GFP complex but not in the negative control (GFP), indicating that TCS1 physically associates with tubulins in Arabidopsis.

To further investigate whether TCS1 localizes to cortical microtubules, we conducted live-cell imaging using a functional TCS1-GFP fusion under the control of *TCS1* promoter. As shown in Fig 5D–5F, TCS1-GFP localizes to puncta along cortical microtubules (mCherry-TUB6) in pavement cells, indicating that TCS1 binds to the microtubules. We then investigated the co-localization of TCS1-GFP and microtubules in developing trichomes. We have previously showed that it is difficult to observe the signal of mCherry labeled-microtubules in trichomes [21]. We therefore used *pTCS1*:*TCS1-GFP* and *GFP-TUB6* -expressing lines to compare TCS1 with microtubules. In *GFP-TUB6* trichomes, transverse microtubule arrays formed rings encircling the elongating branches, without the signal at the extreme apex (Fig 5G) [21]. Similarly, we observed that TCS1-GFP was present in elongating trichome branches, but leave a TCS1-depleted zone at the extreme apex (Fig 5H). These results indicate that TCS1 and microtubules exhibit similar organization patterns in trichomes, further suggesting that TCS1 is a microtubule-binding protein.

As TCS1 directly interacts with microtubules, we asked whether TCS1 could affect microtubule assembly. We therefore added various concentrations of MBP-TCS1 (0, 0.25, 0.5 and 1 μM) and 1 μM MBP to a 20 μM tubulin solution, and tubulin polymerization was investigated by measuring turbidity. As shown in Fig 5I, the presence of MBP-TCS1 increased turbidity, indicating that MBP-TCS1 increases microtubule mass. The assembly rate of tubulins was increased in a dosage-dependent manner with the addition of MBP-TCS1. To confirm this result, we observed the assembly of rhodamine-labeled tubulins incubated with MBP and MBP-TCS1 under confocal microscopy. As shown in Fig 5J, the assembly of microtubules was detected in the presence of MBP-TCS1 rather than MBP. Taken together, these results indicate that TCS1 promotes microtubule assembly.

### TCS1 physically interacts with KCBP/ZWI *in vitro* and *in vivo*

**Fig 6:**
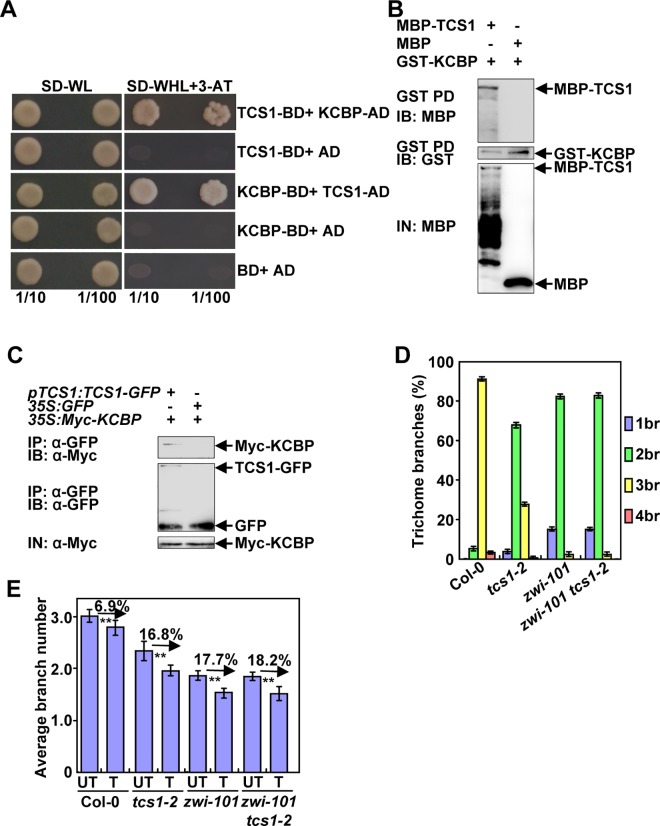
TCS1 physically and genetically interacts with KCBP to control the number of trichome branches. (A) TCS1 interacts with KCBP in yeast cells. (B) TCS1 physically interacts with KCBP *in vitro*. MBP-TCS1 was pulled down (PD) by GST-KCBP immobilized on Glutathione Sepharose 4B and analyzed by immunoblotting (IB) using an anti-MBP antibody. MBP was used as a negative control. (C) TCS1 interacts with KCBP *in vivo*. Total proteins from *pTCS1*:*TCS1-GFP;35SMyc-KCBP* and *35S*:*GFP; 35SMyc-KCBP* plants were immunoprecipitated with GFP-Trap-A (IP), and the immunoblots (IB) were probed with anti-GFP and anti-Myc antibodies, respectively. Myc-KCBP was detected in the immunoprecipitated TCS1-GFP complex. (D) Trichome branch (br) distribution of Col-0, *tcs1-2*, *zwi-101* and *zwi-101 tcs1-2* first pair of leaves at 15 days after germination (DAG). Values are given as mean ± SE. (E) The average number of Col-0, *tcs1-2*, *zwi-101*, *zwi-101 tcs1-2* trichome branches treated with (T) or without (UT) 20 μM oryzalin for 2 hours. The branch number of Col-0, *tcs1-2*, *zwi-101*, *zwi-101 tcs1-2* trichomes was examined after a 10-day recovery on ½ MS medium.

To further understand the molecular mechanism of TCS1 in the regulation of trichome branch number, we performed a yeast two-hybrid screen to identify putative TCS1-binding proteins. TCS1 was fused to the GAL4 DNA binding domain (BD) and used as a bait. In this screen, KCBP/ZWI was identified as a putative TCS1-interacting protein. KCBP/ZWI has been shown to affect microtubules and trichome branches [16], suggesting that TCS1 could interact with KCBP/ZWI to control trichome branches. We tested the interactions between TCS1 and the full length KCBP in yeast cells. As shown in Fig 6A, TCS1 interacted with KCBP in a yeast two-hybrid assay. We then investigated the interaction of TCS1 with KCBP using *in vitro* pull-down experiments. TCS1 was expressed as a maltose binding protein (MBP) fusion protein (MBP-TCS1), while KCBP was expressed as a glutathione S-transferase (GST) fusion protein (GST-KCBP). As shown in Fig 6B, MBP-TCS1 bound to GST-KCBP, while the negative control (MBP) did not bind to GST-KCBP. This result indicates that TCS1 physically interacts with KCBP *in vitro*.

We further performed co-immunoprecipitation analysis to investigate the association of TCS1 with KCBP in Arabidopsis. We generated *35S*:*Myc-KCBP* transgenic plants. We crossed the *pTCS1*:*TCS1-GFP* and *35S*:*GFP* transgenic lines with *35S*:*Myc-KCBP* transgenic plants to generate *pTCS1*:*TCS1-GFP;35S*:*Myc-KCBP* and *35S*:*GFP;35S*:*Myc-KCBP* plants, respectively. Total proteins were isolated and incubated with GFP-Trap-A agarose beads to immunoprecipitate TCS1-GFP and GFP. The anti-GFP and anti-Myc antibodies were used to detect immunoprecipitated proteins, respectively. Myc-KCBP was found in the immunoprecipitated TCS1-GFP complex but not in the negative control (GFP) (Fig 6C), indicating that TCS1 physically associates with KCBP in Arabidopsis.

### *TCS1* genetically interacts with *KCBP* to control the number of trichome branches

As TCS1 physically interacts with KCBP, and *tcs1* mutants showed similar trichome branching phenotypes to *kcbp/zwi* mutants, we sought to establish genetic relationships between *TCS1* and *KCBP* in the regulation of trichome branch number. We obtained the *zwi-101* mutant (SALK_017886) harboring the T-DNA insertion in the *KCBP/ZWI* gene (S8 Fig). The full length mRNA of *KCBP* could not be detected in *zwi-101*, suggesting that *zwi-101* is a loss-of-function allele. The *zwi-101* trichomes exhibited the reduced number of branches (Fig 6D), consistent with previous results [16]. We then generated a *zwi-101 tcs1-2* double mutant and investigated its trichome branch number. As shown in Fig 6D and S9 Fig, the branch number of *zwi-101 tcs1-2* double mutant trichomes was comparable to that of *zwi-101* single mutant trichomes, suggesting that *zwi-101* is epistatic to *tcs1-2* with respect to the number of trichome branches. Considering that both TCS1 and KCBP affect the stability of microtubules, we asked whether genetic interactions between *TCS1* and *KCBP* in trichome branch number are related to microtubule stability. We therefore treated 4-day-old seedlings of Col-0, *zwi-101*, *tcs1-2* and *zwi-101 tcs1-2* with 20 μM oryzalin for 2 hours. After a 10-day recovery on ½ MS medium, the number of Col-0, *zwi-101*, *tcs1-2* and *zwi-101 tcs1-2* trichome branches was investigated. After oryzalin treatment, the number of *zwi-101 tcs1-2* trichome branches was similar to that of *zwi-101* trichome branches (Fig 6E). The oryzalin treatment caused a similar decrease in the average number of *zwi-101 tcs1-2* and *zwi-101* trichome branches. These results suggest that *TCS1* acts genetically with *KCBP* to regulate the number of trichome branches by influencing the stability of microtubules.

KCBP was reported to physically interact with AN in yeast cells [18]. The *an* mutants showed the reduced branches of trichomes in leaves [18,19]. We asked whether *TCS1* and *AN* could function in a common pathway to control trichome branches. To test this, we obtained the *an-101* mutant (SALK_026489) harboring the T-DNA insertion in the *AN* gene (S10 Fig). The full length mRNA of *AN* could not be detected in *an-101*, suggesting that *an-101* is a loss-of-function allele. The *an-101* mutant trichomes mainly had one or two branches (S11 Fig), consistent with previous results [18]. We then generated the *an-101 tcs1-2* double mutant and investigated its trichome branches. The number of *an-101 tcs1-2* trichome branches was similar to that of *an-101* trichome branches (S11 Fig), suggesting an epistatic genetic interaction. We further tested whether TCS1 could physically interact with AN. As shown in S12 Fig, TCS1 did not directly interact with AN *in vitro* (S12 Fig).

## Discussion

A fundamental question in developmental biology is how cell shape is controlled. In plants, cell shape is crucial not only for the function of the individual cell, but also for its role in organ shape and size control. However, the genetic and molecular mechanisms that determine cell shape remain largely unknown in plants. In this study, we report that the *TCS1* gene, which encodes a microtubule binding protein with long coiled-coil domains, is required for trichome cell shape in Arabidopsis. TCS1 directly binds to microtubules and promotes microtubule assembly. TCS1 physically and genetically interacts with KCBP/ZWICHEL to regulate the number of trichome branches by influencing microtubule stability. Thus, our findings reveal a novel genetic and molecular mechanism of TCS1 and KCBP in trichome cell shape control.

### TCS1 regulates trichome cell shape by influencing the stability of microtubules

The *tcs1* trichomes showed the reduced branch number (Fig 1), although *tcs1* plants appear to be similar to wild-type plants. Trichome branching is a complicated process, which is regulated by a number of factors. In Arabidopsis, DNA replication (endoreduplication) in trichome cells influences the number of trichome branches [22,27,28]. However, mutations in *TCS1* did not affect ploidy levels in leaves and nuclear size in trichome cells (S1 Fig). Thus, it is unlikely that TCS1 regulates trichome branch number by influencing DNA replication events. After endoreduplication, a cytoskeleton-dependent polarization event happens during trichome morphogenesis [23], resulting in a total of three to four branches in the mature trichome on leaves. Molecular-genetic and pharmacological studies have established that microtubules are essential for trichome branching in Arabidopsis [23,29]. Interestingly, the trichomes of *tcs1* were hypersensitive to the microtubule-disrupting drug oryzalin in comparison with those of the wild type (Fig 2A and 2B). Similarly, *tcs1* hypocotyls were more sensitive to oryzalin than wild-type hypocotyls (Fig 2E, 2F, and S2 Fig). By contrast, the microtubule-stabilizing drug taxol treatment partially rescued the branch number of *tcs1* trichomes (Fig 2C and 2D). These results suggest that *TCS1* may affect the stability of microtubules, which are crucial for trichome cell morphogenesis. Consistent with this notion, we observed that microtubules in *tcs1* cells disappeared faster than those in wild-type cells when treated with oryzalin (Fig 3 and S3 Fig). Thus, these results support that mutations in *TCS1* influence the stability of microtubules, resulting in the altered trichome cell shape in Arabidopsis.

### TCS1 is a microtubule-binding protein and promotes microtubule assembly

The *TCS1* gene encodes a coiled-coil domain-containing protein, which belongs to a family of long coiled-coil protein that consists of 7 members in Arabidopsis [25]. However, the biological functions of the TCS1 family members are totally unknown in Arabidopsis [25]. Therefore, TCS1 is a novel regulator of trichome cell shape in Arabidopsis. Sequence analyses show that TCS1 homologs are plant-specific proteins (S5 Fig), suggesting that TCS1 and its homologs might have evolved to regulate cell shape in plants. Expression of *TCS1* was detected in all tested tissues (S6 Fig), although the only visible phenotype in *tcs1* mutants was found in trichomes. It is possible that TCS1 might function redundantly with other proteins to influence cell growth in other tissues or cell types.

Several microtubule binding proteins have been known to influence the branch number of trichomes in Arabidopsis [30,31]. Our biochemical analyses showed that TCS1 physically interacts with microtubules *in vitro* and *in vivo* (Fig 5A–5C). Live-cell imaging assay found that TCS1 directly bound to the microtubule in Arabidopsis cells (Fig 5D–5F). In addition, TCS1-GFP and GFP-TUB6 showed similar organization patterns in elongating trichome branches (Fig 5G and 5H). These results support that TCS1 is a microtubule binding protein. It is possible that TCS1 directly binds to microtubules during trichome development and stabilizes microtubules, thereby influencing the formation of trichome branches in Arabidopsis. Biochemical analyses showed that TCS1 promotes microtubule assembly, consistent with the function of TCS1 in stabilizing microtubules (Fig 5I and 5J). The microtubule assembly has been known to influence trichome branch number. For example, mutations in the TUBULIN FOLDING COFACTOR (TCF) C and TCFA result in the unbranched trichome phenotype [13,14]. These mutants were proposed to affect the making of assembly component α/β tubulin dimmers and possibly decrease the assembly of new microtubules. Mutations in *KINESIN-13A*, which promotes microtubule depolymerization, resulted in the increased number of trichome branches [15,32]. Thus, it is possible that TCS1 promotes microtubule assembly and increases the stability of microtubules, thereby influencing trichome branch number in Arabidopsis.

### A possible genetic and molecular mechanism of TCS1 in regulating the number of trichome branches

KCBP, a microtubule motor, regulates cell division and trichome cell shape in Arabidopsis [16,17]. Trichomes on *zwi* leaves had one or two branches with blunt tips. It has been suggested that KCBP participates in the trichome morphogenesis by regulating the local reorientation and stability of microtubules [30,31]. Similarly, TCS1 regulates trichome branch number by influencing the stability of microtubules. The *tcs1* mutants showed similar trichome branch number phenotype to *kcbp/zwi* mutants, suggesting that TCS1 could genetically interact with KCBP to control the branch number of trichomes. Consistent with this idea, our genetic analyses show that *zwi* is epistatic to *tcs1* with respect to trichome branch number. Further results demonstrated that TCS1 physically interacted with KCBP *in vitro* and *in vivo* (Fig 6A–6C). As both TCS1 and KCBP influence the stability of microtubules, it is possible that TCS1 functions with KCBP to control trichome branch number by affecting the dynamics and stability of microtubules in Arabidopsis. Supporting this notion, *zwi-101 tcs1-2* and *zwi-101* trichomes showed a similar level of hypersensitivity to oryzalin (Fig 6E). A recent study have shown that KCBP interacts with both microtubules and actin cytoskeleton to regulate trichome branching and elongation in Arabidopsis [21]. The *zwi* trichomes had the reduced number of branches, shortened stalks and stunted branches [16]. The reduced number of branches in *zwi* trichomes is likely caused by defects in microtubules. By contrast, the transverse cortical F-actin cap at the trichome branch apex has been proposed to regulate polarized branch elongation and tip sharpening [21]. *tcs1* mutants only affected the trichome branch number and had normal stalks and trichome branch tips (Fig 1A and 1B), suggesting that KCBP and TCS1 may have the overlapped function in the regulation of microtubule cytoskeleton rather than actin cytoskeleton.

Genetic studies suggested that KCBP may interact with multiple factors, such as SUZ1, SUZ2 and SUZ3 and might function as a complex, although the *SUZ* genes have not been cloned in Arabidopsis [20]. KCBP has been shown to physically interact with a plant-specific protein kinase termed KCBP-interacting protein kinase (KIPK), a calcium binding protein (KIC) and AN [17,18,33]. KIC regulates trichome morphogenesis by influencing microtubule binding and microtubule-stimulated ATPase activities of KCBP, although the genetic interactions between KCBP and KIC remain unknown [17]. AN is also required for normal trichome morphogenesis in Arabidopsis although AN has been suggested to indirectly regulate microtubules [18,19]. It has been proposed that the level of each protein in the KCBP complex is likely to be crucial for trichome morphogenesis [17]. As TCS1 physically and genetically interacted with KCBP, it is possible that TCS1 and other members in the KCBP complex may have genetic interactions in trichome branching. Supporting this notion, we found an epistatic interaction between *AN* and *TCS1* with respect to the number of trichome branches, although TCS1 does not physically interact with AN (S12 Fig). It will be a worthwhile challenge to build up the genetic and molecular interactions between TCS1 and other members of the KCBP complex in the future. Taken together, our findings reveal a novel genetic and molecular mechanism by which TCS1 interacts with KCBP to control trichome cell shape by influencing the stability of microtubules.

## Materials and Methods

### Plant materials and growth conditions

The *tcs1-1* (SAIL_403_D02), *tcs1-2* (SALK_040648), *tcs1-3* (SALK_078664), *zwi-101* (SALK_017886), and *an1-101* (SALK_026489) mutants were obtained from the Nottingham Arabidopsis Stock Centre (NASC). The T-DNA insertions were verified by PCR and sequencing using the primers described in S1 Table. Arabidopsis seeds were sterilized with 100% isopropanol for 2 min and 10% NaClO (v/v) for 10 min and then washed six times with sterile water. Arabidopsis seeds were dispersed on ½ Murashige and Skoog (MS) medium containing 0.9% agar and 1% glucose and then stored at 4°C for 3 days in the darkness. Plants were grown at 22°C under long-day conditions (a 16-h-light /8-h-dark cycle). To observe etiolated hypocotyls, we grow plants in dark at 22°C.

### Construction and plant transformation

A PCR-based Gateway system was used to generate *35S*:*TCS1*, *pTCS1*:*TCS1-GFP* and *pTCS1*:*GUS* constructs. The *TCS1* CDS was amplified using the primers TCS1-CDS-LP and TCS1-CDS-RP (S1 Table). PCR product was subcloned into the *pCR8/GW/TOPO TA* cloning vector (Invitrogen) using TOPO enzyme. The *TCS1* CDS was then subcloned into the Gateway binary vector *pMDC32* to generate the *35S*:*TCS1* construct. The *TCS1* genomic sequence containing a 2012-bp promoter sequence and 3298bp gene was amplified using the primers gTCS1-GFP-LP and gTCS1-GFP-RP. PCR products were firstly cloned into the *pCR8/GW/TOPO TA* cloning vector (Invitrogen) using TOPO enzyme. The *TCS1* genomic sequence was then subcloned into the *pMDC107* vector to generate the construct *pTCS1*:*TCS1-GFP*. The 2164bp promoter sequence of *TCS1* was amplified using the primers TCS1pro-LP and TCS1pro-RP. PCR products were cloned into the *pCR8/GW/TOPO TA* cloning vector (Invitrogen) using TOPO enzyme. The *TCS1* promoter was then subcloned into the *pMDC164* vector to generate the transformation plasmid *pTCS1*:*GUS*. The plasmids *35S*:*TCS1*, *pTCS1*:*TCS1-GFP* and *pTCS1*:*GUS* were transferred into *tcs1-2* or Col-0 plants using Agrobacterium GV3101, and medium with hygromycin (30μg/mL) was used to select transgenic plants.

### Morphological and cellular analysis

Trichome branches on the first pair of Col-0 and *tcs1-1* leaves were counted at 15 days after germination (DAG). Leaves and etiolated hypocotyls of wild-type and *tcs1-1* mutants were fixed in a solution (formalin, acetic acid, ethanol and H_2_O in a ratio of 1: 0.5: 4.75: 3.75) for 24 hours, dehydrated with a graded ethanol series and dried at critical point in liquid CO_2_. Samples were coated with gold and then observed in an S-4160 Field Emission Scanning Electron Microscope (SEM) (Hitachi).

To determine the effect of *TCS1* on cortical microtubules, the microtubule-disrupting drug oryzalin (3,5-dinitro-N4, N4-dipropylsulfanilamide; Sigma-Aldrich) was applied to trichomes and hypocotyl epidermal cells of the wild type (Col-0) and *tcs1-1* for specific times. The microtubule-stabilizing drug taxol (paclitaxel, Sigma-Aldrich) was used to treat trichomes of the wild type and *tcs1-1*.

To quantify the numbers of cortical microtubules in trichome and hypocotyl cells, the ImageJ software was employed. A line of fixed length (10 μm or 20 μm) perpendicular to the orientation of the most cortical microtubules was drawn, and the number of cortical microtubules across the line was counted. At least 10 cells from each treatment were used, and four lines of fixed length were drawn for each cell. The average number of cortical microtubules before and after treatments was calculated. The Student’s test was used to analyze the significance of the difference.

### GUS staining

Samples (*pTCS1*:*GUS*) were putted into a GUS staining solution [0.1% Nonidet P-40, 1 mM 5-bromo-4-chloro-3-indolyl-b-D-glucuronic acid, 10 mM EDTA, 100 mM Na_3_PO_4_ buffer, and 3 mM each K_3_Fe(CN)_6_/K_4_Fe(CN)_6_] and incubated at room temperature for 6 hours. After GUS staining, 70% ethanol was used to remove chlorophyll.

### Confocal microscopy observation

GFP fluorescence in cells of trichomes and hypocotyls was detected using a Zeiss LSM710 META confocal microscope. GFP was observed using wave lengths of 510 to 530 nm. To study the co-localization of TCS1 and microtubules, we crossed *pTCS1*:*TCS1-GFP* transgenic plants with *mCherry-TUB6* expressing plants. Seeds were germinated on ½ Murashige and Skoog (MS) medium supplemented with 0.9% agar and 1% glucose. Leaves of 6-day-old *mCherry-TUB6;pTCS1*:*TCS1-GFP* seedlings were observed under a spinning disk confocal microscope equipped with lasers for GFP and mCherry (Intelligent Design).

### RNA isolation and semiquantitative RT-PCR analysis

Leaves, stems, cotyledons and roots from 12-day-old seedlings were collected to isolate total RNAs using an RNeasy Plant Mini kit (TIANGEN). Reverse transcription (RT)-PCR was performed using Superscript III reverse transcriptase (Invitrogen). *ACTIN2* mRNA was an internal control. The specific primers used for RT-PCR are shown in S1 Table.

### Yeast two-hybrid assays

The coding sequence of *TCS1* was cloned into *Not*I and *Sal*I sites of the bait vector *pDBleu* (Invitrogen) and the prey vector *pEXP-AD502* (Invitrogen) to generate *TCS1-BD* and *TCS1-AD* constructs, respectively. The specific primers for *TCS1-BD* and *TCS1-AD* were TCS1-Y2H-*Sal*I-LP and TCS1-Y2H-*Not*I-RP (S1 Table). The coding sequence of *KCBP* was cloned into *Not*I and *Sal*I sites of the bait vector *pDBleu* (Invitrogen) and the prey vector *pEXP-AD502* (Invitrogen) to generate *KCBP-BD* and *KCBP-AD* constructs. The specific primers for *KCBP-BD* and *KCBP-AD* constructs were KCBP-Y2H-*Sal*I-LP and KCBP-Y2H-*Not*I-RP (S1 Table). The prey and bait plasmids were co-transformed into the yeast strain PT69-4A to investigate their interactions.

### *In vitro* protein-protein interaction

The coding sequence of *TCS1* was cloned into the vector *pMAL-C2* to generate the *MBP-TCS1* construct. The specific primers for the *MBP-TCS1* construct were MBP-TCS1-LP and MBP-TCS1-RP (S1 Table). The coding sequence of *KCBP* was cloned into the vector *pGEX-4T-1* (Amersham-Pharmacia) to generate the *GST-KCBP* construct. The specific primers for the *GST-KCBP* constructs were GST-KCBP-LP and GST-KCBP-RP (S1 Table). The coding sequence of *AN* was subcloned into the vector *pET-NT* to generate the *AN-His* construct. The specific primers for the *AN-His* construct were AN-His-LP and AN-His-RP (S1 Table). The *MBP-TCS1*, *GST-KCBP* and *AN-His* plasmids were transformed and expressed in *E*. *coli* Rosetta (DE3).

To investigate protein-protein interaction, we performed the pull-down assay. Bacterial lysates containing ~15 μg of MBP-TCS1 or MBP-TCP14 fusion proteins were mixed with ~20 μg of tubulins. Bacterial lysates containing ~15 μg of MBP-TCS1 or MBP proteins were mixed with `~20 μg of AN-His fusion proteins. Amylose resin (30 μL; New England Biolabs) was added into each combined solution with gently rocking at 4°C for 1 h. Bacterial lysate containing ~20 μg of GST-KCBP was mixed with lysate containing ~15 μg of MBP-TCS1 or MBP. Glutathione Sepharose 4B (30 μL;GE Healthcare) was added into each combined solution with gently rocking at 4°C for 1 h. The TGH buffer (1× Complete Protease Inhibitor cocktail [Roche], 150 mM NaCl, 10% glycerol, 1.5 mM MgCl_2_, 50 mM HEPES, pH 7.5, 1 mM phenylmethylsulfonyl fluoride, 1 mM EGTA, pH 8.0, and 1% Triton X-100) was used to wash beads for five times. The isolated proteins were then analyzed by 10% SDS-polyacrylamide gels and determined by immunoblot analysis with anti-MBP, anti-tubulin, anti-GST, and anti-His antibodies (Abmart), respectively.

### Assays of microtubule cosedimentation and assembly

For the microtubule cosedimentation assay, different concentrations of MBP-TCS1 were added to paclitaxel-stabilized microtubules in the PEMT buffer (1 mM MgCl2, 1 mM EGTA, 100 mM PIPES, and 20 μM taxol, pH 6.9). After incubation at 25°C for 30 min, the samples were centrifuged at 100,000g at 25°C for 30 min to separate supernatants and pellets. They were then analyzed by 10% SDS-PAGE and determined by staining the gels with Coomassie Brilliant Blue R 250.

For the microtubule polymerization assay, different concentrations of MBP-TCS1 were added to 20 μM tubulin solution in the PEM buffer (1 mM MgCl2, 1 mM EGTA, 1 mM GTP, and 100 mM PIPES, pH6.9). The polymerization was investigated turbidimetrically by absorbance at 350 nm with a 0.4-cm path quartz cell at 37°C in a DU-640 spectrophotometer (Beckman Coulter, Fullerton, CA). 1 μM MBP was used as a negative control. The data was recorded from time 0 to 35min, when the turbidity in all samples did not increase any more.

For the observation of microtubule assembly, 1μM MBP-TCS1, 20μM Rhodamine labeled tubulins and 1mM GTP were incubated at 37°C for 30 min. The microtubule polymerization was then stopped using 1% glutaraldehyde. Spinning-Disc Confocal Microscopy Imaging was performed on an Olympus IX81 inverted microscope equipped with a Yokogawa spinning-disc confocal head (Yokogawa Electric) and an Andor iXon charge-coupled device camera (Andor Technology). 1μM MBP was used as a negative control. Images were captured using Andor iQ software, version 1.1 (Andor Technology), and processed using ImageJ software.

### Co-immunoprecipitation

The coding sequence of *KCBP* was cloned into the *Xma*I and *Spe*I sites of the *pCAMBIA1300-221-Myc* vector to generate the transformation plasmid *35S*:*Myc-KCBP*. The specific primers used for *35S*:*Myc-KCBP* construct were MYC-KCBP-*Xma*I-LP and MYC-KCBP-*Spe*I-RP (S1 Table). The *35S*:*Myc-KCBP* plasmid was transferred into *tcs1-2* plants using Agrobacterium GV3101, and hygromycin (30μg/mL)-containing medium was used to select transformants. Total proteins from *pTCS1*:*TCS1-GFP;35S*:*Myc-KCBP* and *35S*:*GFP;35S*:*Myc-KCBP* were isolated with the extraction buffer (1× Complete protease inhibitor cocktail, 50 mM Tris/HCl, pH 7.5, 2% Triton X-100, 1 mM EDTA, 150 mM NaCl, 20 mg/mL MG132, and 20% glycerol) and mixed with GFP-Trap-A (Chromotek) for 1 h at 4°C. Beads were washed four times with the wash buffer (1×Complete protease inhibitor cocktail, 50mMTris/HCl, pH7.5, 150mM NaCl, and 0.1% Triton X-100). The immunoprecipitates were analyzed by 10% SDS-polyacrylamide gel and determined by immunoblot analysis with anti-GFP (Abmart) and anti-Myc (Abmart) antibodies, respectively.

### Accession numbers

Arabidopsis Genome Initiative locus identifiers for the genes mentioned in this article are as follows: AT1G19835 (*TCS1*), AT5G65930 (*KCBP*), and AT1G01510 (*AN*).

## Supporting Information

S1 Fig *TCS1* does not affect endoreduplication. (A) The size of nuclei in wild-type Col-0 and *tcs1-1* trichomes. The nuclei were stained by DAPI. (B) The average area of nuclei in Col-0 and *tcs1-1* trichomes. (C) Nuclear DNA ploidy distribution of cells in Col-0 and *tcs1-1* first pair of leaves measured at 13 days after germination (DAG). Values (B and C) are given as mean ± SE. Bars = 20 μm in (A).
(PDF)

S2 Fig Hypocotyl cells of *tcs1-1* are hypersensitive to the microtubule-disrupting drug oryzalin. (A) Scanning electron microscope images of Col-0 and *tcs1-1* cells in the top and middle regions of etiolated hypocotyls grown in ½ MS for 15 days in dark. Bars = 200 μm. (B) Scanning electron microscope images of Col-0 and *tcs1-1* cells in the top and middle regions of etiolated hypocotyls grown in ½ MS containing 0.3 μM oryzalin for 15 days in dark. Bars = 200 μm. (C) The average length of epidermal cells in the middle regions of Col-0 and *tcs1-1* hypocotyls treated with oryzalin. Col-0 and *tcs1-1* seedlings were grown in ½ MS containing 0, 0.25 μM and 0.3 μM oryzalin (OZ) for 15 days in dark. (D) The average width of epidermal cells in the middle regions of Col-0 and *tcs1-1* hypocotyls treated with oryzalin. Col-0 and *tcs1-1* seedlings were grown in ½ MS containing 0, 0.25 μM and 0.3 μM oryzalin (OZ) for 15 days in dark. Values (C and D) are given as mean ± SE. **P<0.01 compared with the wild type (Student’s *t* test).
(PDF)

S3 Fig Microtubules in epidermal cells of *tcs1-1* cotyledons are hypersensitive to the microtubule-disrupting drug oryzalin. Cortical microtubules in epidermal cells of *GFP-TUB6* and *GFP-TUB6;tcs1-1* cotyledon veins treated with 5 μM oryzalin for 10 minutes. Bars = 20 μm.
(PDF)

S4 Fig Identification of the *TCS1* gene. (A) PCR identification of the T-DNA insertion in *tcs1-1* with T-DNA specific primers (LB1) and flanking primers (LP and RP). (B) PCR identification of the T-DNA insertion in *tcs1-2* with T-DNA specific primers (LBa1) and flanking primers (LP and RP). (C) PCR identification of the T-DNA insertion in *tcs1-3* with T-DNA specific primers (LBa1) and flanking primers (LP and RP). (D) RT-PCR analysis of *TCS1* expression in Col-0, *tcs1-1*, *tcs1-2* and *tcs1-3* seedlings. RT-PCR was performed on first-strand cDNA prepared from 2-week-old seedlings. cDNA was standardized by reference to an *ACTIN2* standard. (E) The average trichome branch number of Col-0, *tcs1-1*, *pTCS1*:*TCS1-GFP;tcs1-1#1* and *pTCS1*:*TCS1-GFP;tcs1-1#2* first pair of leaves at 15 days after germination (DAG). Values (E) are given as mean ± SE. **P<0.01 compared with the wild type (Student’s *t* test).
(PDF)

S5 Fig Phylogenetic tree of TCS1 and its homologs in different species. The phylogenetic tree was constructed using the neighbor-joining method of the MEGA6 program (http://www.megasoftware.net/mega.html). Values at nodes represent percentages of 1000 bootstrap replicates. The scale bar at the bottom represents the genetic distance.
(PDF)

S6 Fig Expression of the *TCS1* gene. RT-PCR analysis of *TCS1* expression in roots, flowers, 10-day-old seedlings, rosette leaves and cauline leaves.
(PDF)

S7 Fig Quantification of the binding affinity of TCS1 and AUG8 with microtubules. (A) MBP-TCS1 fusion protein was cosedimented with paclitaxel-stabilized microtubules (5 μM). After high-speed centrifugation, the amount of MBP-TCS1 in pellets increased when higher concentrations of MBP-TCS1 proteins were added before reaching saturation. (B) His-AUG8 fusion protein was cosedimented with paclitaxel-stabilized microtubules (5 μM). After high-speed centrifugation, the amount of His-AUG8 in pellets increased when higher concentrations of His-AUG8 proteins were added before reaching saturation. (C) Quantification of the binding affinity of TCS1 with microtubules shown in (A) compared with that of AUG8 with microtubules shown in (B). The binding of TCS1 and AUG8 to microtubules was saturated at a stoichiometry of about 0.38 M MBP-TCS1 and 0.22 M His-AUG8 per mole of tubulin dimers, respectively.
(PDF)

S8 Fig Identification of the *zwi-101* mutant. (A) The insertion of T-DNA in *zwi-101* (SALK_017886) is shown. (B and C) PCR identification of the T-DNA insertion in *zwi-101* with T-DNA specific primers (LBa1) and flanking primers (LP and RP). (D) Expression levels of *KCBP* in Col-0 and *zwi-101* seedlings as determined by RT-PCR.
(PDF)

S9 Fig *zwi-101* is epistatic to *tcs1-2* with respect to trichome branch number. (A) The average number of Col-0, *tcs1-2*, *zwi-101* and *zwi-101 tcs1-2* trichome branches of the first pair of leaves at 15 days after germination (DAG). (B) Scanning electron microscope images of Col-0, *tcs1-2*, *zwi-101* and *zwi-101 tcs1-2* trichome branches of first pair of leaves at 15 days after germination (DAG). Values (A) are given as mean ± SE. **P<0.01 compared with the respective controls (Student’s *t* test). Bars = 100 μm.
(PDF)

S10 Fig Identification of the *an-101* mutant. (A) The insertion of T-DNA in *an-101* (SALK_026489) is shown. (B and C) PCR identification of the T-DNA insertion in *an-101* with T-DNA specific primers (LBa1) and flanking primers (LP and RP). (D) Expression levels of *AN* in Col-0 and *an-101* seedlings as determined by RT-PCR.
(PDF)

S11 Fig *an-101* is epistatic to *tcs1-2* with respect to trichome branch number. (A) The average number of Col-0, *tcs1-2*, *an-101* and *an-101 tcs1-2* trichome branches of the first pair of leaves at 15 days after germination (DAG). (B) Scanning electron microscope images of Col-0, *tcs1-2*, *an-101* and *an-101 tcs1-2* trichome branches of the first pair of leaves at 15 days after germination (DAG). Values (A) are given as mean ± SE. Bars = 100 μm.
(PDF)

S12 Fig TCS1 does not physically interact with AN. AN-His proteins were pulled down (PD) by MBP-TCS1 immobilized on amylose resin and analyzed by immunoblotting (IB) using an anti-His antibody. MBP was used as a negative control.
(PDF)

S1 Table List of primers used in this study (PDF)
