# Supplementary figures and images for: TCS1, a Microtubule-Binding Protein, Interacts with KCBP/ZWICHEL to Regulate Trichome Cell Shape in Arabidopsis thaliana
Source: PLoS Genet. 2016 Oct 21;12(10):e1006266. doi: 10.1371/journal.pgen.1006266 (PMC5074588; doi:10.1371/journal.pgen.1006266)

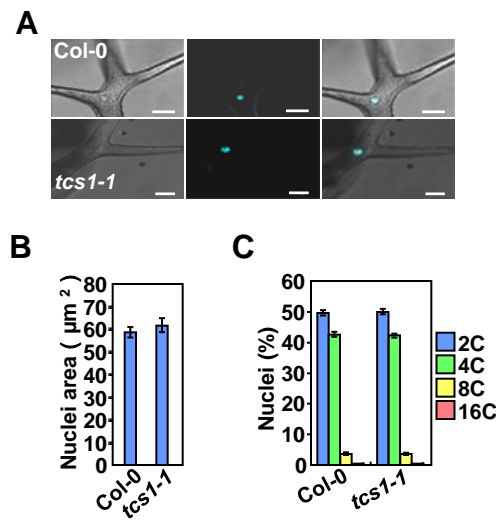

**Figure S1**

Supplement: S1 Fig — (A) The size of nuclei in wild-type Col-0 and tcs1-1 trichomes. The nuclei were stained by DAPI. (B) The average area of nuclei in Col-0 and tcs1-1 trichomes. (C) Nuclear DNA ploidy distribution of cells in Col-0 and tcs1-1 first pair of leaves measured at 13 days after germination (DAG). Values (B and C) are given as mean ± SE. Bars = 20 μm in (A). (PDF) [file pgen.1006266.s001.pdf]

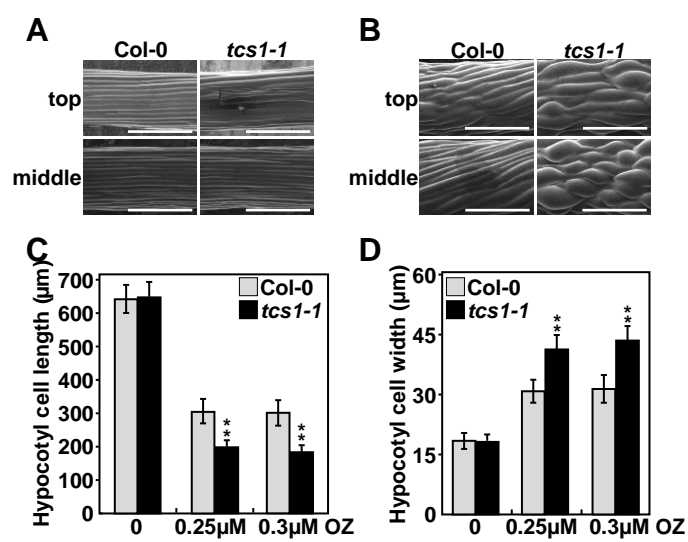

Figure S2

Supplement: S2 Fig — (A) Scanning electron microscope images of Col-0 and tcs1-1 cells in the top and middle regions of etiolated hypocotyls grown in ½ MS for 15 days in dark. Bars = 200 μm. (B) Scanning electron microscope images of Col-0 and tcs1-1 cells in the top and middle regions of etiolated hypocotyls grown in ½ MS containing 0.3 μM oryzalin for 15 days in dark. Bars = 200 μm. (C) The average length of epidermal cells in the middle regions of Col-0 and tcs1-1 hypocotyls treated with oryzalin. Col-0 and tcs1-1 seedlings were grown in ½ MS containing 0, 0.25 μM and 0.3 μM oryzalin (OZ) for 15 days in dark. (D) The average width of epidermal cells in the middle regions of Col-0 and tcs1-1 hypocotyls treated with oryzalin. Col-0 and tcs1-1 seedlings were grown in ½ MS containing 0, 0.25 μM and 0.3 μM oryzalin (OZ) for 15 days in dark. Values (C and D) are given as mean ± SE. **P<0.01 compared with the wild type (Student’s t test). (PDF) [file pgen.1006266.s002.pdf]

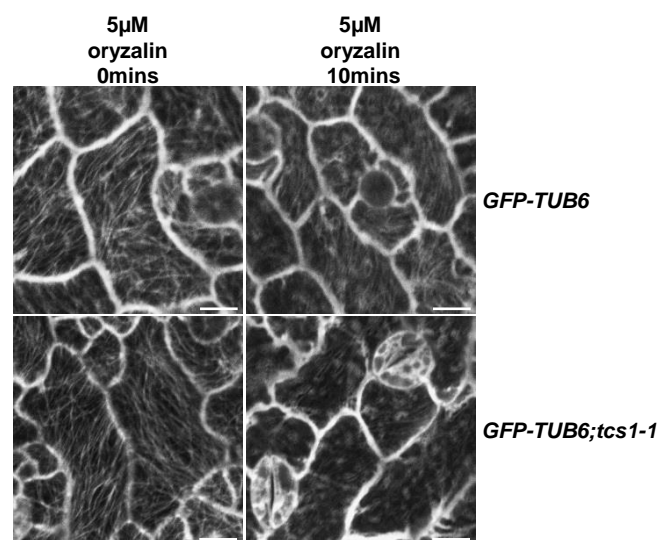

**Figure S3**

Supplement: S3 Fig — Cortical microtubules in epidermal cells of GFP-TUB6 and GFP-TUB6;tcs1-1 cotyledon veins treated with 5 μM oryzalin for 10 minutes. Bars = 20 μm. (PDF) [file pgen.1006266.s003.pdf]

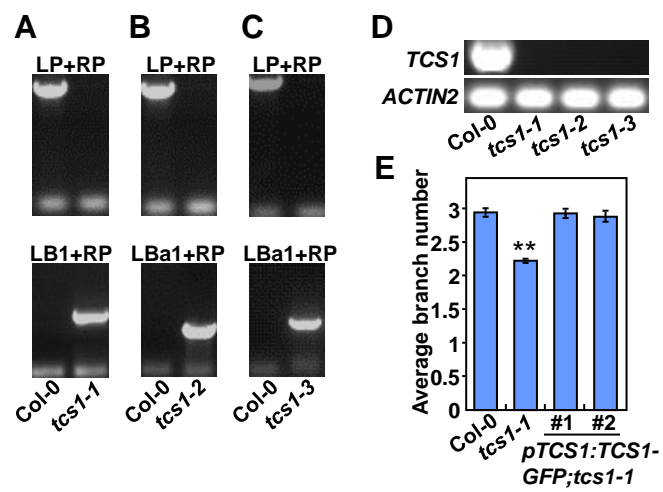

Figure S4

Supplement: S4 Fig — (A) PCR identification of the T-DNA insertion in tcs1-1 with T-DNA specific primers (LB1) and flanking primers (LP and RP). (B) PCR identification of the T-DNA insertion in tcs1-2 with T-DNA specific primers (LBa1) and flanking primers (LP and RP). (C) PCR identification of the T-DNA insertion in tcs1-3 with T-DNA specific primers (LBa1) and flanking primers (LP and RP). (D) RT-PCR analysis of TCS1 expression in Col-0, tcs1-1, tcs1-2 and tcs1-3 seedlings. RT-PCR was performed on first-strand cDNA prepared from 2-week-old seedlings. cDNA was standardized by reference to an ACTIN2 standard. (E) The average trichome branch number of Col-0, tcs1-1, pTCS1:TCS1-GFP;tcs1-1#1 and pTCS1:TCS1-GFP;tcs1-1#2 first pair of leaves at 15 days after germination (DAG). Values (E) are given as mean ± SE. **P<0.01 compared with the wild type (Student’s t test). (PDF) [file pgen.1006266.s004.pdf]

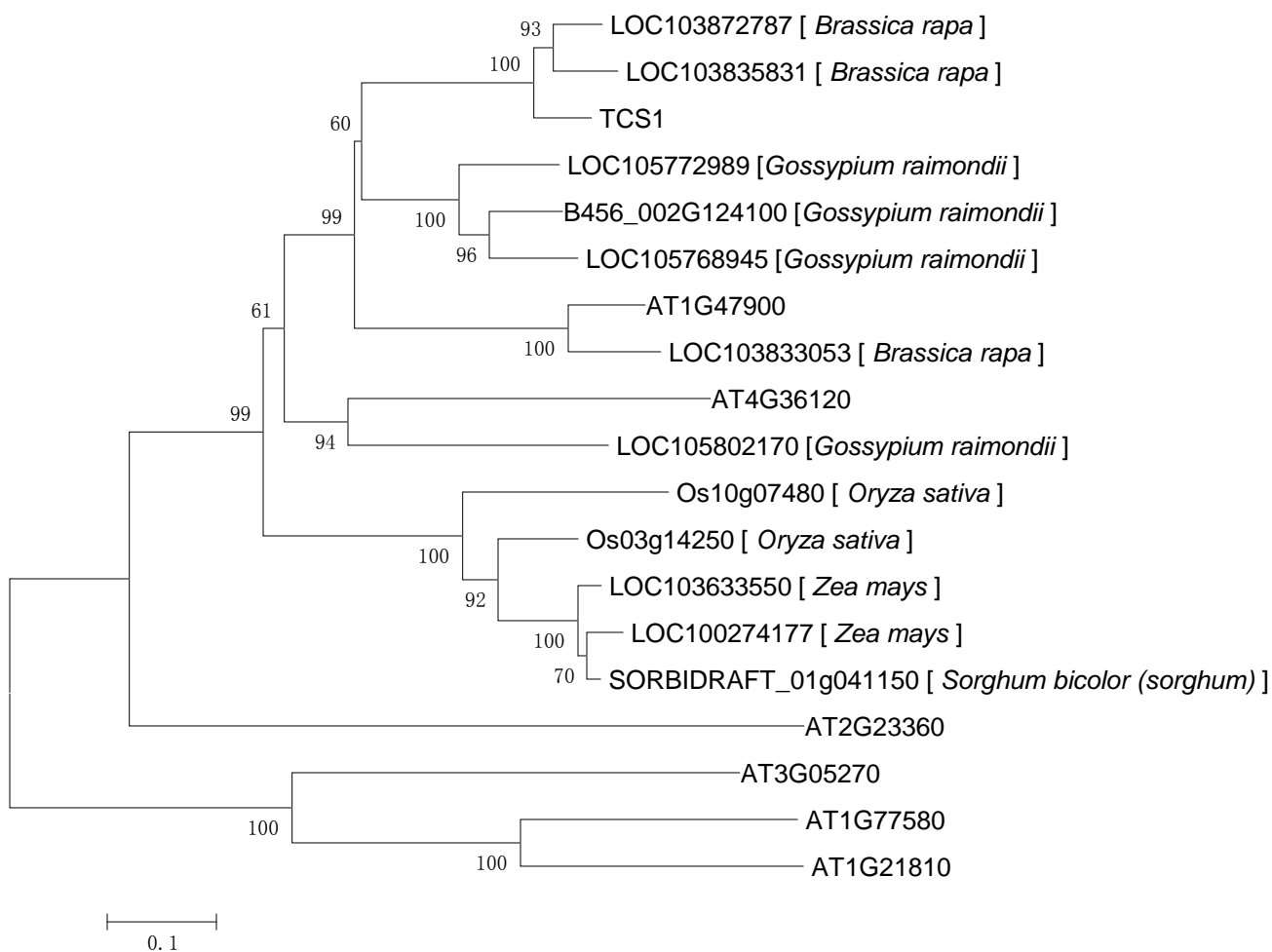

**Figure S5**

Supplement: S5 Fig — The phylogenetic tree was constructed using the neighbor-joining method of the MEGA6 program (http://www.megasoftware.net/mega.html). Values at nodes represent percentages of 1000 bootstrap replicates. The scale bar at the bottom represents the genetic distance. (PDF) [file pgen.1006266.s005.pdf]

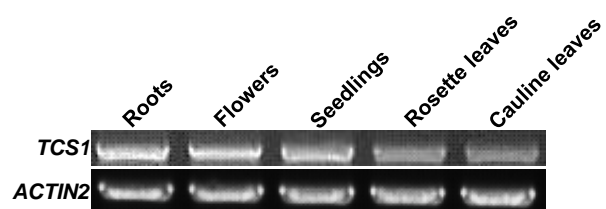

Figure S6

Supplement: S6 Fig — RT-PCR analysis of TCS1 expression in roots, flowers, 10-day-old seedlings, rosette leaves and cauline leaves. (PDF) [file pgen.1006266.s006.pdf]

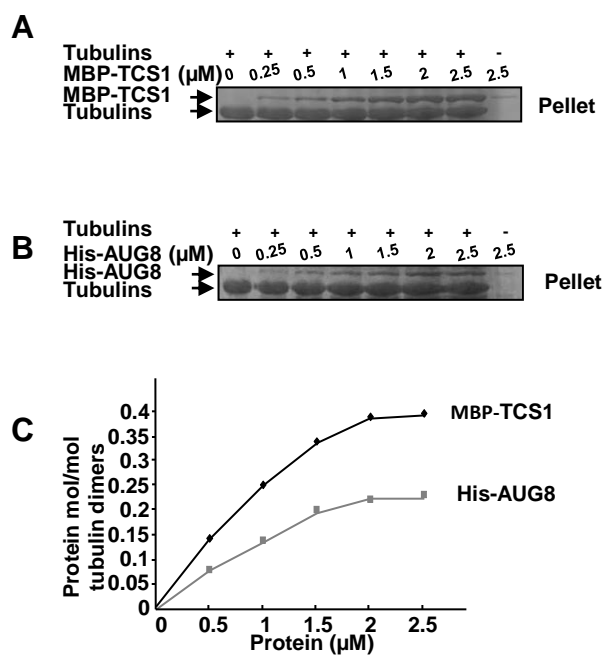

Figure S7

Supplement: S7 Fig — (A) MBP-TCS1 fusion protein was cosedimented with paclitaxel-stabilized microtubules (5 μM). After high-speed centrifugation, the amount of MBP-TCS1 in pellets increased when higher concentrations of MBP-TCS1 proteins were added before reaching saturation. (B) His-AUG8 fusion protein was cosedimented with paclitaxel-stabilized microtubules (5 μM). After high-speed centrifugation, the amount of His-AUG8 in pellets increased when higher concentrations of His-AUG8 proteins were added before reaching saturation. (C) Quantification of the binding affinity of TCS1 with microtubules shown in (A) compared with that of AUG8 with microtubules shown in (B). The binding of TCS1 and AUG8 to microtubules was saturated at a stoichiometry of about 0.38 M MBP-TCS1 and 0.22 M His-AUG8 per mole of tubulin dimers, respectively. (PDF) [file pgen.1006266.s007.pdf]

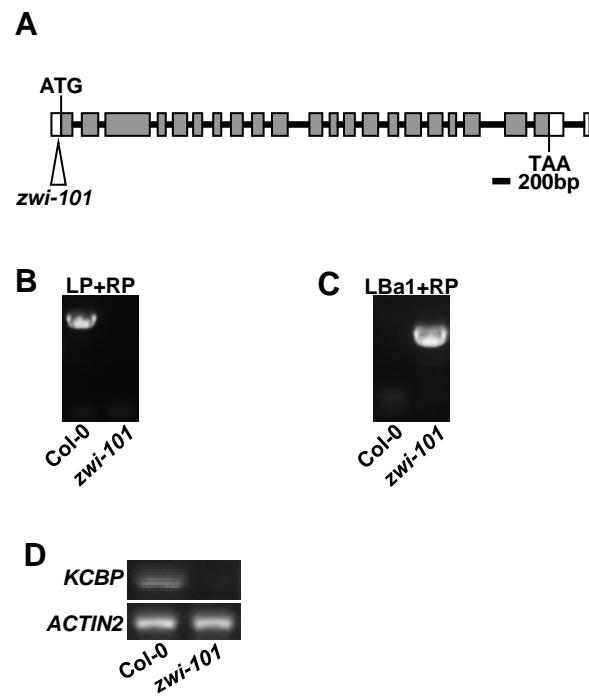

Figure S8

Supplement: S8 Fig — (A) The insertion of T-DNA in zwi-101 (SALK_017886) is shown. (B and C) PCR identification of the T-DNA insertion in zwi-101 with T-DNA specific primers (LBa1) and flanking primers (LP and RP). (D) Expression levels of KCBP in Col-0 and zwi-101 seedlings as determined by RT-PCR. (PDF) [file pgen.1006266.s008.pdf]

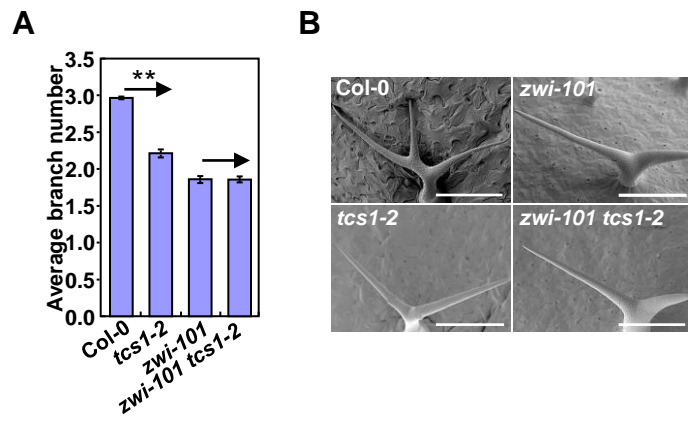

Figure S9

Supplement: S9 Fig — (A) The average number of Col-0, tcs1-2, zwi-101 and zwi-101 tcs1-2 trichome branches of the first pair of leaves at 15 days after germination (DAG). (B) Scanning electron microscope images of Col-0, tcs1-2, zwi-101 and zwi-101 tcs1-2 trichome branches of first pair of leaves at 15 days after germination (DAG). Values (A) are given as mean ± SE. **P<0.01 compared with the respective controls (Student’s t test). Bars = 100 μm. (PDF) [file pgen.1006266.s009.pdf]

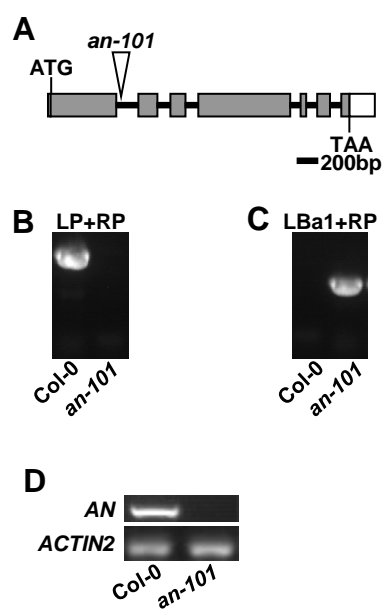

Figure S10

Supplement: S10 Fig — (A) The insertion of T-DNA in an-101 (SALK_026489) is shown. (B and C) PCR identification of the T-DNA insertion in an-101 with T-DNA specific primers (LBa1) and flanking primers (LP and RP). (D) Expression levels of AN in Col-0 and an-101 seedlings as determined by RT-PCR. (PDF) [file pgen.1006266.s010.pdf]

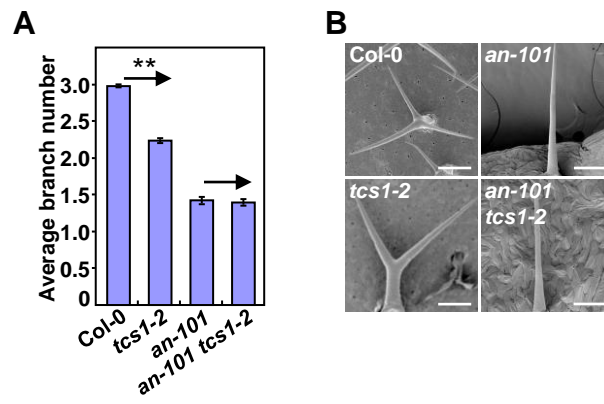

Figure S11

Supplement: S11 Fig — (A) The average number of Col-0, tcs1-2, an-101 and an-101 tcs1-2 trichome branches of the first pair of leaves at 15 days after germination (DAG). (B) Scanning electron microscope images of Col-0, tcs1-2, an-101 and an-101 tcs1-2 trichome branches of the first pair of leaves at 15 days after germination (DAG). Values (A) are given as mean ± SE. Bars = 100 μm. (PDF) [file pgen.1006266.s011.pdf]

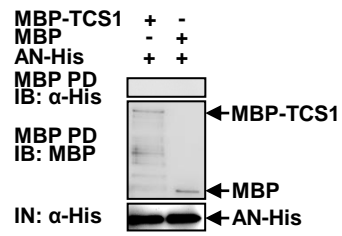

**Figure S12**

Supplement: S12 Fig — AN-His proteins were pulled down (PD) by MBP-TCS1 immobilized on amylose resin and analyzed by immunoblotting (IB) using an anti-His antibody. MBP was used as a negative control. (PDF) [file pgen.1006266.s012.pdf]
