# Supplementary material for: TCS1, a Microtubule-Binding Protein, Interacts with KCBP/ZWICHEL to Regulate Trichome Cell Shape in Arabidopsis thaliana
Source: PLoS Genet. 2016 Oct 21;12(10):e1006266. doi: 10.1371/journal.pgen.1006266 (PMC5074588; doi:10.1371/journal.pgen.1006266)
Supplement: S1 Table — (PDF) [file pgen.1006266.s013.pdf]

Supplemental Table 1. List of primers used in this study

Name	Primers
**Primers for verifying T-DNA**	
SAIL_403_D02-LP	TTCTGAGATTCCGGATGATTG
SAIL_403_D02-RP	GGAGAAGAACCTGCTGAATCC
SALK_040648-LP	TCAGAAGTCTAATGGCATGGG
SALK_040648-RP	TATGAGACGGTGTAAGTGGGC
SALK_078664-LP	TCAGAAGTCTAATGGCATGGG
SALK_078664-RP	TATGAGACGGTGTAAGTGGGC
SALK_017886-LP	GAGGCGTAGATCGATCAGATG
SALK_017886-RP	AGGATGATCTAAAGCTTCCGC
SALK_026489-LP	TCCTTCACCTGCAATGAAAAC
SALK_026489-RP	TAGGTCTCCGACTTGTCCATG
**Primers for constructs**	
TCS1-CDS-LP	GATCGTAAGAGTTGGCCATGGAAG
TCS1-CDS-RP	TTACTTTGCTTTAGTGGAGAAGAAC
TCS1pro-LP	GAGCTCCCGTGGAATTGCTATTTACAGACTG
TCS1pro-RP	CCATGGCTTCATAATCAACAAACAATTCAAC
gTCS1-GFP-LP	GGGGACAAGTTTGTACAAAAAAGCAGGCTAGCATATGACCAAA ACTCAGATTTG
gTCS1-GFP-RP	GGGGACCACTTTGTACAAGAAAGCTGGGTCCTTTGCTTTAGTGG AGAAGAAC
MYC–KCBP-*Xma*I-LP	TCCCCCCGGGTTGAGGGCCAACGAGGCAGTAATT
MYC–KCBP-*Spe*I-RP	CTAGACTAGTTCAACTATCTGCCTCATCTTTTCGT
MBP-TCS1-LP	ggatccGATCGTAAGAGTTGGCCATGGAAG
MBP-TCS1-RP	gtcgacTTACTTTGCTTTAGTGGAGAAGAACCT
GST-KCBP-LP	ACGCGTCGACTCGAGGGCCAACGAGGCAGTAATT
GST-KCBP-RP	AAGGAAAAAAGCGGCCGCTCAACTATCTGCCTCATCTTTTCGT
AN-HIS-LP	cagattacgctggatccgaattcAGCAAGATCCGTTCGTCTGCGACA
AN-HIS-RP	GGTGGTGGTGGTGGTGCTCGAGATCGATCCAACGTGTGATACCA TC
MBP-TCP14-LP	GAATTCCAAAAGCCAACATCAAGTATCTTAAAT
MBP-TCP14-RP	AAGCTTGCCTAATCTTGCTGATCCTCCTCATCACCAC
TCS1-Y2H-*Sal*I-LP	ACGCgtcgaccGATCGTAAGAGTTGGCCATGG
TCS1-Y2H-*Not*I-RP	AAGGAAAAAAgcggccgcTTACTTTGCTTTAGTGGAGAAGAAC
KCBP-Y2H-*Sal*I-LP	ACGCGTCGACCGAGGGCCAACGAGGCAGTAATT
KCBP-Y2H -*Not*I-RP	AAGGAAAAAAGCGGCCGCTCAACTATCTGCCTCATCTTTTCGT
**Primers for RT-PCR**	
TCS1-RT-FP	ATGGATCGTAAGAGTTGGCCATGGAAG
TCS1-RT-RP	TTACTTTGCTTTAGTGGAGAAGAACCT
KCBP-RT-LP	ATGGAGGGCCAACGAGGCAG
KCBP-RT-RP	TCAACTATCTGCCTCATCTT
AN-RT-LP	ATGAGCAAGATCCGTTCGTC

AN-RT-RP	TTAATCGATCCAACGTGTGA
ACTIN2-FP	GAAATCACAGCACTTGCACC
ACTIN2-RP	AAGCCTTTGATCTTGAGAGC
